# Development of a Canadian socioeconomic status index for the study of health outcomes related to environmental pollution

**DOI:** 10.1186/s12889-015-1992-y

**Published:** 2015-07-28

**Authors:** Emily Chan, Jesus Serrano, Li Chen, David M. Stieb, Michael Jerrett, Alvaro Osornio-Vargas

**Affiliations:** Department of Pediatrics, University of Alberta, Edmonton, Canada; School of Public Health, University of Alberta, Edmonton, Canada; Population Studies Division, Health Canada, Ottawa, Canada; Population Studies Division, Health Canada, Vancouver, Canada; School of Public Health, University of California, Berkeley, USA

**Keywords:** Socioeconomic status, Environment, Health

## Abstract

**Background:**

Socioeconomic status (SES) is an important determinant of health and potential modifier of the effects of environmental contaminants. There has been a lack of comprehensive indices for measuring overall SES in Canada. Here, a more comprehensive SES index is developed aiming to support future studies exploring health outcomes related to environmental pollution in Canada.

**Methods:**

SES variables (*n* = 22, Census Canada 2006) were selected based on: cultural identities, housing characteristics, variables identified in Canadian environmental injustice studies and a previous deprivation index (Pampalon index). Principal component analysis with a single varimax rotation (factor loadings ≥ │60│) was performed on SES variables for 52974 census dissemination areas (DA). The final index was created by averaging the factor scores per DA according to the three components retained. The index was validated by examining its association with preterm birth (gestational age < 37 weeks), term low birth weight (LBW, <2500 g), small for gestational age (SGA, <10 percentile of birth weight for gestational age) and PM_2.5_ (particulate matter ≤ 2.5 μm) exposures in Edmonton, Alberta (1999–2008).

**Results:**

Index values exhibited a relatively normal distribution (median = 0.11, mean = 0.0, SD = 0.58) across Canada. Values in Alberta tended to be higher than in Newfoundland and Labrador, Northwest Territories and Nunavut (Pearson chi-square p < 0.001 across provinces). Lower quintiles of our index and the Pampalon’s index confirmed know associations with a higher prevalence of LBW, SGA, preterm birth and PM_2.5_ exposure. Results with our index exhibited greater statistical significance and a more consistent gradient of PM_2.5_ levels and prevalence of pregnancy outcomes.

**Conclusions:**

Our index reflects more dimensions of SES than an earlier index and it performed superiorly in capturing gradients in prevalence of pregnancy outcomes. It can be used for future research involving environmental pollution and health in Canada.

**Electronic supplementary material:**

The online version of this article (doi:10.1186/s12889-015-1992-y) contains supplementary material, which is available to authorized users.

## Background

Reports such as the Canada Health Survey [1] and the Canadian Community Health Survey [2] indicated that inequalities of health resulting from socioeconomic status (SES) required urgent scrutiny [3]. Because the majority of health data is released in area-level form in comparison to individual-level form as a result of privacy concerns, geographical proxies, where the SES for small areas is linked to health data from administrative databases are often utilized [3]. Most of these studies have used neighbourhood income as the indicator of social disparity and mortality as the health indicator [3]. Measuring SES using a single indicator, however, is unlikely to completely reflect its complexity. Deprivation indices including other measures such as unemployment, social class, income, marital status, occupation, and education have been developed for Great Britain [4], Spain [5], and Italy [6].

Until recently, only two deprivation indices for Canada have been developed, each with a specific purpose. Matheson et al. [7] proposed an index called the “Can-Marg” using Census 2006 data, in which they focussed on examining inequalities in health and other social problems. Four deprivation criteria: residential instability, material deprivation, dependency and ethnic concentration were defined and inequalities in 18 health and behavioural problems from the Canadian Community Health Survey (CCHS) reported [7]. However, the index that is mostly used in Canadian research has been the Pampalon index, developed in Quebec. Pampalon et al. illustrated its value by linking it to overall Canadian premature mortality rates in 2001 [3]. The group developed their index based on Townsend’s definition of deprivation [8] and included variables such as education and marital status. More specifically, their index was divided into two components: social and material. The Pampalon index only included six variables in the analyses: employment, income, education, marital status, single parent family, and living alone, while the Canadian Census form from which the index was developed, contains over 200 variables.

Among other factors like “individual susceptibility” (e.g. genetic polymorphisms), environmental stressors such as radiation, chemicals, and viruses, as well as dietary habits, psycho-social stress, and social characteristics are known to contribute to the occurrence of common childhood conditions. There recently has been growing interest in environmental injustice, a concept suggesting that those populations with lower SES may be vulnerable to greater exposure to environmental pollutants than their higher SES counterparts, and consequently experiencing potentially increased health risks. Building on this concept, the U.S. Institute of Medicine coined the term “double jeopardy” to emphasize the combined risk often faced by socially disadvantaged groups. Specifically, groups experiencing higher environmental exposure are often more susceptible because they have higher rates of smoking, obesity, poor nutrition, and adverse occupational exposures [9].

Thus a need exists for a comprehensive index of socioeconomic status that is indicative of the Canadian population, which can be used for research involving environmental pollution and health outcomes. For that purpose, we aimed to develop a novel SES index that is comprehensive and more encompassing of the Canadian population, by incorporating cultural identities, examining factors relevant to health outcomes from environmental pollution, and considering other variables used in previous environmental injustice studies.

## Methods

### Data extraction

Socioeconomic data were analyzed from the Canadian Census 2006. For our evaluation, we extracted Census data from CANSIM, Canada’s socioeconomic database which provides free access to a range of the latest statistics [10]. The Census was completed on May 16, 2006 and 32.5 million people were included. One in every five households received a long questionnaire with 53 questions in comparison to 8 for the short form [11]. Here, we used data from the long questionnaire forms. These data cover all of Canada’s dissemination areas (DAs), which are small regions consisting of 400 to 700 people [12]. Canada has 52974 DAs, ranging from 34 for Nunavut to 18923 for Ontario.

### Variables

A set of 22 variables from the 2006 Census was selected based on: (1) cultural identities [13]; (2) potential environmental pollutants related to health outcomes [14, 15]; (3) Canadian environmental injustice studies [16–18]; and (4) variables utilized in the deprivation index for Canada proposed by Pampalon [3] (Table 1). Studies in the United States have indicated a clear relationship between several racial groups with regards to SES [19–21]. In an effort to investigate the phenomenon in Canada, we grouped the cultural identities reported in the census as the individual’s ancestry, based on four categories from the “Human Developmental Index” (HDI): origins from (1) very high sum; (2) high sum; (3) medium sum; and (4) low sum countries [13]. The HDI takes into account the human development of a country and ranks them according to life expectancy, literacy, education and standards of living [13]. We included a category for those with aboriginal identities based on responses to the “Indian Status” and “Aboriginal identities” question on the Census and combined those who were North American Indian, Metis, Inuit, multiple Aboriginal identities, and aboriginal responses not included elsewhere. Each of the variables was expressed as proportions per dissemination area (DA). Variables obtained as raw counts of the answers to questions were converted to proportions by dividing by the number of people answering the question. Since the data used in the creation of the index were collected from questions answered with the long form of the Census questionnaire, the proportions were based on the variables corresponding to 20 % of the population of Canada. Employment rate, median income and prevalence of low income after taxes were not transformed since they were originally reported as proportions per DA in the census database.

**Table 1:** Parameters and variables used in the selection for PCA analysis

Parameter (number of candidate variables)	Variable (census 2006)
Cultural identities (*n* = 5)	Very high sum human developmental index (HDI); high HDI; medium HDI; low HDI; aboriginal group status
Potential existence of indoor environmental pollutants related to health outcomes (*n* = 4)	Construction of homes before 1946; 1946–1970; 1971–1990; 1991–2006
Environmental injustice indicators (*n* = 7)	Marital status; prevalence of low income after taxes; car, truck, or van for commute; public transit, walking or bicycling for commute; multiple family households; owning a home; renting accommodations
Variables utilized in a previously proposed deprivation index for Canada (*n* = 6)	Educational certificate; no educational certificate; employment rate; median income; total lone-parent families; divorced or widowed status

Lastly, we incorporated a variable that we thought was important for health outcomes related to environmental pollution: age of the home (construction of homes before 1946, 1946–1970, 1971–1990, 1991–2006) as a proxy for age of the neighbourhood. In the United States, it has been shown that older neighbourhoods are more likely to have lead paint [15], asbestos [14] and have more infiltration of fine particles from outdoors to indoors [22].

### Construction of SES index

Principal component analysis (PCA) with a single varimax rotation (factor loadings ≥ │60│) was performed on the selected 22 Census socioeconomic variables (SAS 9.2, North Carolina, USA). The analyses were completed for all DAs of Canada whereupon we utilized two criteria for the selection of components: (1) Kaiser Criterion (eigenvalues ≥1); and (2) individual proportion of variances per component explaining ≥ 10 % of the overall variability. The final SES index was created by averaging the factor scores (a numerical representation of the linear relationship between variables and the components) per DA, according to the three components retained.

### Validation of our SES index

#### Adverse birth outcomes

We attempted to validate our index by utilizing the well-researched concept that low SES may be related to adverse birth outcomes [23, 24]. Here, data on all singleton live births between 1999 and 2008 in Edmonton were accessed through Statistics Canada (Additional file 1). Pregnancy outcomes under study were preterm birth (gestational age < 37 weeks), term low birth weight (LBW, <2500 g), and small for gestational age (SGA, <10 percentile of birth weight for gestational age). Spearman correlations and t-tests were used to assess associations between the index and pregnancy outcomes.

#### Particulate matter (PM _2.5_)

We also evaluated another known [25], but less explored association between our SES index and concentrations of particulate matter with a mean aerodynamic diameter < 2.5 μm (PM_2.5_). Spearman correlation and t-tests were used to examine relationships between PM_2.5_ exposures and SES indices. PM_2.5_ exposures were assigned by mapping the mother’s six-character postal code to a monthly surface PM_2.5_ concentration, based on a North American land use regression model that incorporated observations from fixed-site monitoring stations and satellite-derived estimates of PM_2.5_. Exposures were estimated for the entire duration of pregnancy. Methods are described in detail elsewhere [26].

#### Comparison of Chan index to Pampalon index

We compared the association of our SES index and that of Pampalon’s [3] with adverse birth outcomes and PM_2.5_ concentrations using Spearman correlations and t-tests. The Pampalon index is a commonly used SES index in Canada that was developed using variables from the 2006 Census with: (1) known relations to health; (2) past use as geographical proxies; (3) past utilizations with the material or social dimensions of deprivation; (4) availability by DA [3]. PCA was used on the variables and two components were found that are now used as the Pampalon indices: Values for the Material and Social components. The Pampalon index value used to validate our index were accessed through their website [3, 27]. Both indices represent the Canadian SES situation in 2006 and comparisons assume the same similar Canada wide SES distribution around the year of 2006.

## Results

### Principal component analysis (PCA)

Three components were extracted for Canada, with a cumulative retained variation of 58.9 %. Each component yielded different conceptual meanings when variables were placed together, based on a preconceived categorical variable classification (Table 2). Component 1 contained variables related to: (1) social advantages, and (2) high material ownership; (Additional file 2). Component 2 included variables related to economic advantages, (Additional file 3) and Component 3 (Additional file 4) was entirely different in composition and direction and contained variables indicative of being: (1) socially disadvantaged and having (2) specific cultural identities. Interestingly, aboriginal status was not included in the cultural identities of Component 3, but instead medium sum HDI groups were incorporated. Additionally, age of the home was not included in any of the components. For this analysis, the final SES index was obtained averaging the components retained by utilizing the formula [C1 + C2+ (−1*C3)]/3. Given the disadvantage connotation of the variables included in Component 3, we multiplied factor scores for Component 3 by −1 to achieve a comprehensive index for Canada, which would integrate all components for an overall meaning of “socioeconomic status”.

**Table 2:** List of new variables (Chan et al., 2015) created for analyses of components and their descriptors

New variable (*n* = 8)	Census variables (*n* = 22)
1) High material ownership	Home ownership
	Car, truck or van for commute
2) Low material ownership	Rent accommodation
	Public transportation use
3) Socially advantaged	Marital status
	One family households
4) Economically advantaged	Employment rate
	Median income
	Certificate, diploma or degree
5) Socially disadvantaged	Single, widowed or divorced
	Multiple family households
	Lone parent families
6) Economically disadvantaged	Prevalence of low income after taxes
	No certificate, diploma or degree
7) Indication of potential children’s environmental hazard	Construction of home ≤1946 to 1970
	Construction of home 1971–1990
	Construction of home 1991–2006
8) Cultural identities	Very high sum HDI
	High sum HDI
	Medium sum HDI
	Low sum HDI
	Aboriginal

An overall examination of the index for all of Canada shows a relatively normal distribution (median = 0.11, mean = 0.0, standard deviation = 0.58). However, an individual analysis of the distribution of indices within each province and territory per DA, according to the Canada wide index, yielded different results. Here, the SES index distribution for Canada was divided into quintiles and the number of DAs within each quintile per province and territory was investigated. (Additional file 5) Alberta showed increasing numbers of DAs within higher values of the SES index, while Newfoundland, Northwest Territories, and Nunavut showed greater numbers of DAs within lower values of the SES index. A chi-square test examining the distribution of DAs within each quintile of SES showed that it was not homogenous among provinces (Pearson chi-square = 2637.9, p < 0.001).

**Fig. 1:**
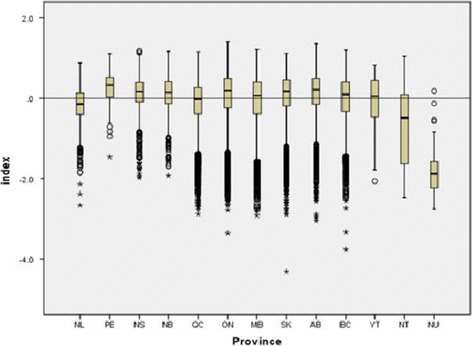
Boxplot distribution of median SES index by province and territory (*n* = 13). Whiskers represent upper and lower range, while asterisks represent outliers. Bottom and top of boxes are the first and third quartiles, while the center line represents medians. (NL = Newfoundland and Labrador, PE = Prince Edward Island, NS = Nova Scotia, NB = New Brunswick, QC = Quebec, ON = Ontario, MB = Manitoba, SK = Saskatchewan, AB = Alberta, BC = British Columbia, YT = Yukon, NT = Northwest Territories, NU = Nunavut)

Furthermore, boxplots of the distribution of SES index by province and territory showed more obvious similarities and trends in outliers (Fig. 1). Here, all provinces are mostly grouped together, but Nunavut and the Northwest Territories show the majority of DAs are lower than the country’s average SES index. The mean SES index for the rest of the provinces and territories was slightly above average, with the exception of Newfoundland and Labrador. Additionally, very obvious low SES index outliers were seen in Saskatchewan, British Columbia and Ontario.

### Validation of SES index

#### Prevalence of adverse pregnancy outcomes and PM_2.5_ exposure

**Fig. 2:**
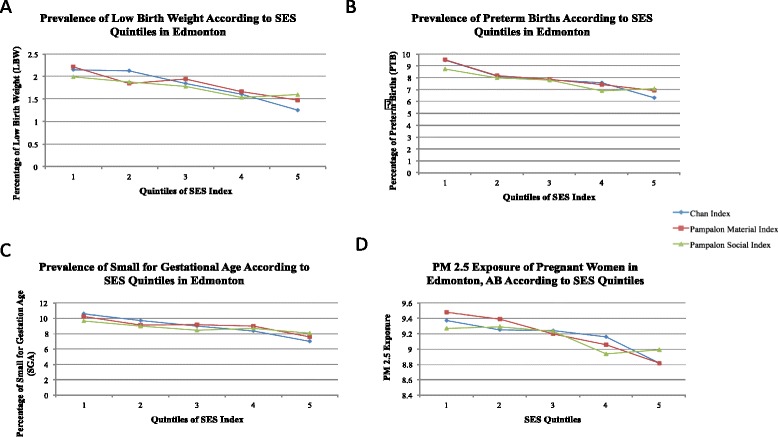
Comparison of the prevalence of low birth weight (panel **a**), preterm births (panel **b**), small for gestational age (panel **c**), and PM _2.5_ exposures (panel **d**) according to Chan et al. and Pampalon et al. indices

Lower quintiles in our SES index were significantly (p < 0.0001) associated with increasing prevalence of LBW, preterm birth and SGA in Edmonton (Fig. 2). This was corroborated with significantly lower mean SES indices for LBW (−0.227), preterm (−0.211) and SGA (−0.216) infants compared to normal weight (−0.138), term (−0.140) and appropriate for gestational age (−0.138) infants (p < 0.0001).

Lower quintiles of our SES index were also significantly associated with higher exposure to PM_2.5_ (p < 0.0001) (Fig. 2).

#### Comparisons of our SES index and Pampalon’s index

Both material and social components of Pampalon’s deprivation indices behaved similarly to our SES index when examining prevalence of LBW, PTB and SGA and PM_2.5_ exposure (Fig. 2). However, there was a more consistent gradient in prevalence of LBW, preterm birth and SGA by quintile of our index compared to the Pampalon indices, more noticeable in the case of LBW (p < 0.0001 vs. p < 0.01). Conversely, there was a more consistent gradient in PM_2.5_ by quintile of the Pampalon material deprivation index compared to our index and the social deprivation index. Correlations between PM_2.5_ and the three indices were very similar (Chan index: r = −0.11, p < 0.0001, Pampalon Material index: r = −0.15, p < 0.0001, Pampalon Social index: r = −0.15, p < 0.0001).

## Discussion

Although our SES index is not the first to be developed for Canada, it likely reflects more fully the dimensions of SES in Canada for purposes of examining health outcomes from environmental pollution. While our index similarly includes aspects of social and material deprivation, it is novel in that we explored the contribution of: 1) age of homes as a proxy for age of neighbourhood which may in turn be an indicator of potential indoor environmental pollution; and 2) cultural identities with special attention to First Nations groups. Additionally, since our index is comprised of a single scale unlike the Pampalon indices, it is more easily communicated and better suited for presenting data directed toward studies investigating health outcomes and environmental pollution, for instance using maps.

More specifically, we examined the age of the homes as a proxy for the age of the neighbourhood. Older neighbourhoods more likely contain asbestos [14], lead paint [15] or increased indoor infiltration of fine outdoor particles [22]. Interestingly, age of the homes was not included in any of the three components for our SES index. This observation may be explained by Canada’s relatively strong social programs, which may have weakened correlations between older homes and living in poverty as seen in the United States. For example, advances geared toward the development of newer government subsidized accommodations in an effort to decrease poverty have been in place with programs such as the Newfoundland and Labrador Poverty Reduction Strategy [28]. Another explanation may include a possible trend in Canada toward middle class or wealthy populations living in older, more established neighbourhoods and homes. This may also have diluted the relationship seen in the US between inhabiting older homes and living in poverty.

Cultural differences were strongly evident with our SES index. Our index differs from Matheson et al.’s “Can-Marg” in that they utilized visible minority and recent immigration status (within 5 years). We grouped cultural origins by examining “ethnic origins”, which takes into account the ancestry of the Canadian population. This may be a more accurate indication of ethnicity, as recent immigration has mostly been from skilled workers from China, who generally have higher SES [29]. This effect was illustrated in the “Can-Marg” index, where ethnicity was positively associated with better health outcomes and more healthy behaviours [7]. We also examined “visible minority” and “recent immigration” in the development of our index (data not shown), and these variables were not associated with any of our components. This pattern was also seen in attempts to include ethnicity through “recent immigration” or “visible minorities” by Jerrett et al. [17]. Thus, by utilizing “ethnic origins”, we may be able to overcome this potential characteristic of the population. Inclusion of aboriginal groups with HDI categories in our index was novel. As 4 % of Canada’s population (1.2 million people) in 2006, aboriginal groups in Canada represent the second largest population in a country internationally [30]. Although there is a large population of aboriginals living in Canada (5 % in Alberta, 14 % in Saskatchewan and Manitoba, 85 % in Nunavut, 51 % in the Northwest Territories, 23 % in the Yukon) [30], this variable interestingly did not appear in any of the three components of our SES index. Historically, aboriginal groups (especially in First Nations communities) have low response to the Census and this may be a source of bias in that this population’s responses may be missing. Nonetheless, aboriginal identity is important to consider because aboriginal families are more likely to experience poverty than the overall population of Canada [30]. For example, those with aboriginal cultural identities are more likely than other Canadians to consist of single parent families (50 % of children in census metropolitan areas) [30]. Another explanation for the lack of aboriginal cultural identities contributing to any of the three components of our SES index may include dilution of the relationship with variables associated with poverty, as there may be different definitions of social and economic advantages for aboriginals living in Northern Canada, where aboriginals comprise a large proportion of the population (Yukon: 25 %, NWT: 50.3 %, Nunavut: 83.6 % in 2006). The aspects of “wealth” and “deprivation” could easily be obscured in these areas, as the attainment of education or even the use of a vehicle in comparison to other forms of transportation may be influenced by traditional forms of living.

Because the index is novel, it was important to test its validity against an extensively used index, the Pampalon deprivation index, exploring an outcome for which associations with SES are well documented such as adverse birth outcomes and exposure to PM_2.5_. Adverse birth outcomes with low SES relationships are a heavily researched area and our results showing increasing prevalence of PTB, LBW, and SGA with lower SES corroborate what has been published previously [23, 24]. Additionally, we observed a more consistent gradient of the occurrence of the outcomes with lower values of our index compared to the Pampalon index, while the reverse was true for PM_2.5_ exposures during pregnancy. We established the validity of our index based on several evaluative criteria: 1) demonstrated similar findings to those reported in the literature showing correlations between SES and adverse birth outcomes; 2) showed potential for supporting our hypothesis of environmental injustice in Canada by demonstrating associations of low index values with increased PM_2.5_ exposure; and 3) showed similar, but stronger findings in comparison with an older index. A clear advantage of our index is that it consists of a single value and is therefore simpler to interpret. A limitation to our index is that while a single value may be useful for easier interpretation, the Pampalon index would allow for independent analyses of material and social deprivation for public health policy and intervention purposes. However, another advantage to utilizing our index is that a background in using past indices such as the Townsend index for interpretation of the Pampalon index is also not required. A limitation of working with indices based on Census data in Canada is the lower number of variables collected in the most recent Census [31]. It is also assumed that SES will be stable over time, serving as a proxy in population based studies using data from other years.

## Conclusion

We focussed our efforts on the development of a national index for Canada for purposes of investigating health outcomes and environmental pollution. We found that it performed superiorly to an earlier index in capturing gradients in prevalence of adverse pregnancy outcomes. We intend to use this index to investigate environmental injustice in Canada by applying aggregated geospatial analysis techniques to examine associations of SES and industrial chemical emissions and the incidence of childhood cancer and other pediatric health outcomes in Canada. Lastly, this new index has the potential to enable a better assessment of SES inequalities in a variety of health outcomes related to environmental pollution in Canada at the DA level.

## Additional files

Additional file 1:
**Descriptive table of general characteristics of all birth outcomes in Edmonton.** * In accordance with Statistics Canada disclosure rules, all frequencies were randomly rounded to base five, but percentages are based on unrounded data. Additional file 2:
**Factor loadings (*100) for Canada and its provinces and territories (n=13) corresponding to Component 1.** (AB=Alberta, BC=British Columbia, SK=Saskatchewan, MB=Manitoba, ON=Ontario, QB=Quebec, NB=New Brunswick, NS=Nova Scotia, PEI=Prince Edward Island, NFL=Newfoundland, YK=Yukon, NV=Nunavut, NWT=Northwest Territories). Additional file 3:
**Factor loadings (*100) for Canada and its provinces and territories (n=13) corresponding to Component 2.** (AB=Alberta, BC=British Columbia, SK=Saskatchewan, MB=Manitoba, ON=Ontario, QB=Quebec, NB=New Brunswick, NS=Nova Scotia, PEI=Prince Edward Island, NFL=Newfoundland, YK=Yukon, NV=Nunavut, NWT=Northwest Territories). Additional file 4:
**Factor loadings (*100) for Canada and its provinces and territories (n=13) corresponding to Component 3.** (AB=Alberta, BC=British Columbia, SK=Saskatchewan, MB=Manitoba, ON=Ontario, QB=Quebec, NB=New Brunswick, NS=Nova Scotia, PEI=Prince Edward Island, NFL=Newfoundland, YK=Yukon, NV=Nunavut, NWT=Northwest Territories). Additional file 5:
**Distribution of the percentage of DAs within each quintile of Canada wide SES index according to province and territory (p < 0.001 using Pearson chi-square), absolute numbers of DAs are indicated in brackets.** (AB=Alberta, BC=British Columbia, MB=Manitoba, NB=New Brunswick, NL=Newfoundland and Labrador, NS=Nova Scotia, NT=Northwest Territories, NU=Nunavut, ON=Ontario, PE=Prince Edward Island, QC=Quebec, SK=Saskatchewan, YT=Yukon).
