# Supplementary material for: Development of a Canadian socioeconomic status index for the study of health outcomes related to environmental pollution
Source: BMC Public Health. 2015 Jul 28;15:714. doi: 10.1186/s12889-015-1992-y (PMC4517649; doi:10.1186/s12889-015-1992-y)
Supplement: Additional file 1: — Descriptive table of general characteristics of all birth outcomes in Edmonton. * In accordance with Statistics Canada disclosure rules, all frequencies were randomly rounded to base five, but percentages are based on unrounded data. [file 12889_2015_1992_MOESM1_ESM.docx]

| Variable | Preterm birth  n (%) | Low birth weight  n (%) | Small for gestational age  n (%) | Mean PM_2.5_ (µg/m^3^) |
| --- | --- | --- | --- | --- |
| Sex |  |  |  |  |
| male | 2945 (8.31%) | 480 (1.48%) | 3320 (9.37%) | 9.17 |
| female | 2495 (7.39%) | 665 (2.13%) | 2880 (8.54%) | 9.17 |
| Maternal age |  |  |  |  |
| <18 | 115 (9.66%) | 20 (1.87%) | 110 (9.28%) | 9.4 |
| 18 – 29 | 3265 (7.77%) | 705 (1.82%) | 3910 (9.31%) | 9.2 |
| 30 – 39 | 1950 (7.83%) | 405 (1.76%) | 2080 (8.36%) | 9.11 |
| 40+ | 10 (10.14%) | 15 (1.54%) | 100 (9.26%) | 9.01 |
| Marital status |  |  |  |  |
| Single | 670 (9.38%) | 150 (2.32%) | 770 (10.79%) | 10.01 |
| Married | 3295 (6.90%) | 735 (1.65%) | 4090 (8.58%) | 9.14 |
| Widowed | 0 (0.00%) | 0 (0.00%) | 5 (16.67%) | 9.55 |
| Divorced | 60 (9.76%) | 15 (2.70%) | 75 (12.20%) | 10.11 |
| Separated | 0 (0.00%) | 0 (0.00%) | 0 (0.00%) | 8.01 |
| unknown | 1415 (10.34%) | 245 (2.00%) | 1260 (9.22%) | 8.81 |
| Parity |  |  |  |  |
| 1^st^ birth | 2560 (8.06%) | 605 (2.07%) | 3495 (11.02%) | 9.14 |
| 2^nd^ birth | 1525 (6.69%) | 300 (1.41%) | 1620 (7.11%) | 9.17 |
| 3^rd^ or greater birth | 1350 (9.24%) | 235 (1.77%) | 1075 (7.36%) | 9.22 |
| unknown | 5 (20.00%) | 5 (25.00%) | 5 (20.00%) | 9.81 |
| Birth year |  |  |  |  |
| 1999 | 470 (7.36%) | 100 (1.69%) | 590 (9.23%) | 10.56 |
| 2000 | 495 (7.96%) | 100 (1.75%) | 590 (9.49%) | 9.96 |
| 2001 | 520 (8.12%) | 110 (1.87%) | 605 (9.45%) | 9.22 |
| 2002 | 485 (7.66%) | 110 (1.88%) | 535 (8.45%) | 9.71 |
| 2003 | 560 (8.49%) | 110 (1.82%) | 540 (8.19%) | 9.17 |
| 2004 | 540 (8.08%) | 90 (1.47%) | 575 (8.61%) | 9.83 |
| 2005 | 570 (8.46%) | 105 (1.70%) | 565 (8.40%) | 7.96 |
| 2006 | 565 (7.62%) | 130 (1.90%) | 670 (9.05%) | 8.84 |
| 2007 | 565 (7.00%) | 145 (1.93%) | 750 (9.31%) | 7.87 |
| 2008 | 670 (8.02%) | 145 (1.89%) | 780 (9.35%) | 9.07 |
|  |  |  |  |  |
|  |  |  |  |  |

Supplement 1. Descriptive table of general characteristics of all birth outcomes in Edmonton. * In accordance with Statistics Canada disclosure rules, all frequencies were randomly rounded to base five, but percentages are based on unrounded data.
