# Supplementary material for: Development of a Canadian socioeconomic status index for the study of health outcomes related to environmental pollution
Source: BMC Public Health. 2015 Jul 28;15:714. doi: 10.1186/s12889-015-1992-y (PMC4517649; doi:10.1186/s12889-015-1992-y)
Supplement: Additional file 2: — Factor loadings (*100) for Canada and its provinces and territories (n=13) corresponding to Component 1. (AB=Alberta, BC=British Columbia, SK=Saskatchewan, MB=Manitoba, ON=Ontario, QB=Quebec, NB=New Brunswick, NS=Nova Scotia, PEI=Prince Edward Island, NFL=Newfoundland, YK=Yukon, NV=Nunavut, NWT=Northwest Territories). [file 12889_2015_1992_MOESM2_ESM.docx]

| **Variable** | **Canada** | **AB** | **BC** | **SK** | **MB** | **ON** | **QB** | **NB** | **NS** | **PEI** | **NFL** | **YK** | **NV** | **NWT** |
| --- | --- | --- | --- | --- | --- | --- | --- | --- | --- | --- | --- | --- | --- | --- |
| No certificate, degree or diploma |  |  |  |  |  |  |  |  |  |  |  | -89 | -97 | -88 |
| Certificate, degree or diploma |  |  |  |  |  |  |  |  |  |  |  | 90 | 96 | 90 |
| Employment rate |  |  |  |  |  |  |  |  |  |  |  | 78 | 83 | 88 |
| Median income |  |  |  |  |  |  |  |  |  |  |  |  | 84 | 78 |
| Single, divorced or widowed | -83 | -88 | -80 | 84 | -85 | -86 | -86 | -87 | -92 | -92 | 82 | -66 |  | -74 |
| Married | 84 | 87 | 80 | -82 | 86 | 85 | 90 | 87 | 92 | 93 | -84 | 62 |  | 72 |
| Prevalence of low income after taxes |  |  |  |  |  |  |  |  |  |  | 69 |  |  |  |
| Car, van or truck for commute | 76 | 73 | 79 |  | 77 | 79 |  | 79 | 82 | 71 |  |  | 76 |  |
| Public transit use | -72 | -75 | -79 |  | -77 | -75 |  | -80 | -82 | -73 |  |  | -68 |  |
| Total lone parent families |  |  |  | 83 |  |  |  |  |  | -71 | 72 |  |  |  |
| Own home | 84 | 85 | 87 | -64 | 81 | 82 | 84 | 90 | 90 | 92 | -84 | 64 |  |  |
| Rent accommodation | -80 | -83 | -84 | 66 | -86 | -77 | -84 | -89 | -87 | -90 | 83 |  |  |  |
| Construction of home ≤ 1946 to 1970 |  |  |  |  |  |  |  |  |  |  |  |  |  |  |
| Construction of home 1971-1990 |  |  |  |  |  |  |  |  |  |  |  |  |  |  |
| Construction of home 1991-2006 |  |  |  |  |  |  |  |  |  |  |  |  |  |  |
| One family households | 82 | 81 | 77 |  | 79 | 83 | 78 | 73 | 79 | 77 |  |  |  |  |
| Multiple family households |  |  |  |  |  |  |  |  |  |  |  |  | -65 |  |
| Very high sum HDI |  |  |  |  |  |  |  |  |  |  |  | 85 | 95 | 91 |
| High sum HDI |  |  |  |  |  |  |  |  |  |  |  | 77 | 73 | 76 |
| Medium sum HDI |  |  |  |  |  |  |  |  |  |  |  |  |  | 69 |
| Low sum HDI |  |  |  |  |  |  |  |  |  |  |  |  |  |  |
| Aboriginal |  |  |  | 61 |  |  |  |  |  |  |  | -86 | -96 | -95 |

Supplement 2. Factor loadings (*100) for Canada and its provinces and territories (n=13) corresponding to Component 1. (AB=Alberta, BC=British Columbia, SK=Saskatchewan, MB=Manitoba, ON=Ontario, QB= Quebec, NB=New Brunswick, NS=Nova Scotia, PEI=Prince Edward Island, NFL=Newfoundland, YK=Yukon, NV=Nunavut, NWT=Northwest Territories)
