# Supplementary material for: Development of a Canadian socioeconomic status index for the study of health outcomes related to environmental pollution
Source: BMC Public Health. 2015 Jul 28;15:714. doi: 10.1186/s12889-015-1992-y (PMC4517649; doi:10.1186/s12889-015-1992-y)
Supplement: Additional file 3: — Factor loadings (*100) for Canada and its provinces and territories (n=13) corresponding to Component 2. (AB=Alberta, BC=British Columbia, SK=Saskatchewan, MB=Manitoba, ON=Ontario, QB=Quebec, NB=New Brunswick, NS=Nova Scotia, PEI=Prince Edward Island, NFL=Newfoundland, YK=Yukon, NV=Nunavut, NWT=Northwest Territories). [file 12889_2015_1992_MOESM3_ESM.docx]

| **Variable** | **Canada** | **AB** | **BC** | **SK** | **MB** | **ON** | **QB** | **NB** | **NS** | **PEI** | **NFL** | **YK** | **NV** | **NWT** |
| --- | --- | --- | --- | --- | --- | --- | --- | --- | --- | --- | --- | --- | --- | --- |
| No certificate, degree or diploma | -73 |  | -40 |  | -90 | -84 |  | -87 | -89 | -96 | -91 |  |  |  |
| Certificate, degree or diploma | 86 | 85 | 83 |  | 93 | 88 |  | 88 | 90 | 96 | 91 |  |  |  |
| Employment rate | 72 | 75 | 76 | 73 |  |  |  | 73 | 68 |  | 80 |  |  |  |
| Median income | 71 | 70 | 62 |  | 74 | 65 |  | 70 | 76 |  | 74 |  |  |  |
| Single, divorced or widowed |  |  |  |  |  |  |  |  |  |  |  | -66 |  |  |
| Married |  |  |  |  |  |  |  |  |  |  |  | 62 |  |  |
| Prevalence of low income after taxes |  |  |  |  |  |  |  |  |  |  |  |  |  |  |
| Car, van or truck for commute |  |  |  | 79 |  |  |  |  |  |  |  |  |  |  |
| Public transit use |  |  |  |  |  |  |  |  |  |  |  |  |  | -63 |
| Total lone parent families |  |  |  |  |  |  |  |  |  |  |  |  |  |  |
| Own home |  |  |  | 62 |  |  |  |  |  |  |  | 64 | -89 | 63 |
| Rent accommodation |  |  |  |  |  |  |  |  |  |  |  |  | 90 |  |
| Construction of home ≤ 1946 to 1970 |  |  |  |  |  |  |  |  |  |  |  |  |  |  |
| Construction of home 1971-1990 |  |  |  |  |  |  |  |  |  |  |  |  |  |  |
| Construction of home 1991-2006 |  |  |  |  |  |  |  |  |  |  |  |  |  |  |
| One family households |  |  |  | 82 |  |  |  |  |  |  |  |  | -66 | 81 |
| Multiple family households |  |  |  |  |  |  |  |  |  |  |  |  |  |  |
| Very high sum HDI |  |  |  |  | 63 |  | -75 |  |  |  |  | 85 |  |  |
| High sum HDI |  |  |  |  |  |  | 69 |  |  |  |  | 77 |  |  |
| Medium sum HDI |  |  |  |  |  |  | 79 |  |  |  |  |  |  |  |
| Low sum HDI |  |  |  |  |  |  | 66 |  |  |  |  |  |  |  |
| Aboriginal |  |  |  |  |  |  |  |  |  |  |  | -86 |  |  |

Supplement 3. Factor loadings (*100) for Canada and its provinces and territories (n=13) corresponding to Component 2. (AB=Alberta, BC=British Columbia, SK=Saskatchewan, MB=Manitoba, ON=Ontario, QB= Quebec, NB=New Brunswick, NS=Nova Scotia, PEI=Prince Edward Island, NFL=Newfoundland, YK=Yukon, NV=Nunavut, NWT=Northwest Territories)
