# Supplementary material for: Development of a Canadian socioeconomic status index for the study of health outcomes related to environmental pollution
Source: BMC Public Health. 2015 Jul 28;15:714. doi: 10.1186/s12889-015-1992-y (PMC4517649; doi:10.1186/s12889-015-1992-y)
Supplement: Additional file 4: — Factor loadings (*100) for Canada and its provinces and territories (n=13) corresponding to Component 3. (AB=Alberta, BC=British Columbia, SK=Saskatchewan, MB=Manitoba, ON=Ontario, QB=Quebec, NB=New Brunswick, NS=Nova Scotia, PEI=Prince Edward Island, NFL=Newfoundland, YK=Yukon, NV=Nunavut, NWT=Northwest Territories). [file 12889_2015_1992_MOESM4_ESM.docx]

| **Variable** | **Canada** | **AB** | **BC** | **SK** | **MB** | **ON** | **QB** | **NB** | **NS** | **PEI** | **NFL** | **YK** | **NV** | **NWT** |
| --- | --- | --- | --- | --- | --- | --- | --- | --- | --- | --- | --- | --- | --- | --- |
| No certificate, degree or diploma |  | 64 |  | -81 |  |  | -88 |  |  |  |  |  |  |  |
| Certificate, degree or diploma |  |  |  | 76 |  |  | 91 |  |  |  |  |  |  |  |
| Employment rate |  |  |  |  |  |  |  |  |  |  |  |  |  |  |
| Median income |  |  |  | 64 |  |  | 68 |  |  |  |  |  |  |  |
| Single, divorced or widowed |  |  |  |  |  |  |  |  |  |  |  |  | 89 |  |
| Married |  |  |  |  |  |  |  |  |  |  |  |  | -85 |  |
| Prevalence of low income after taxes |  |  |  |  |  |  |  |  |  |  |  |  |  |  |
| Car, van or truck for commute |  |  |  |  |  |  |  |  |  |  | 82 |  |  |  |
| Public transit use |  |  |  |  |  |  |  |  |  |  | -77 |  |  |  |
| Total lone parent families |  | 61 |  |  |  |  |  |  |  |  |  |  | 67 |  |
| Own home |  |  |  |  |  |  |  |  |  |  |  |  |  | -61 |
| Rent accommodation |  |  |  |  |  |  |  |  |  |  |  |  |  | 68 |
| Construction of home ≤ 1946 to 1970 |  |  |  |  | 94 |  |  | -79 |  | -79 |  |  |  | 82 |
| Construction of home 1971-1990 |  |  |  |  | -86 |  |  | 69 |  |  |  | -77 |  |  |
| Construction of home 1991-2006 |  |  |  |  |  |  |  |  | 70 | 84 |  | 73 |  |  |
| One family households |  |  |  |  |  |  |  |  |  |  |  |  |  |  |
| Multiple family households | 70 |  | 72 |  |  | 69 |  |  |  |  |  |  |  |  |
| Very high sum HDI | -72 |  | -62 |  |  | -85 |  |  | -65 |  |  |  |  |  |
| High sum HDI |  |  |  |  |  |  |  |  |  |  |  |  |  |  |
| Medium sum HDI | 76 |  | 87 |  |  | 81 |  |  |  |  |  |  |  |  |
| Low sum HDI |  |  |  |  |  |  |  |  |  |  |  | 64 |  |  |
| Aboriginal |  | 71 |  |  |  |  |  |  | 72 |  |  |  |  |  |

Supplement 4. Factor loadings (*100) for Canada and its provinces and territories (n=13) corresponding to Component 3. (AB=Alberta, BC=British Columbia, SK=Saskatchewan, MB=Manitoba, ON=Ontario, QB= Quebec, NB=New Brunswick, NS=Nova Scotia, PEI=Prince Edward Island, NFL=Newfoundland, YK=Yukon, NV=Nunavut, NWT=Northwest Territories)
