# Supplementary material for: Development of a Canadian socioeconomic status index for the study of health outcomes related to environmental pollution
Source: BMC Public Health. 2015 Jul 28;15:714. doi: 10.1186/s12889-015-1992-y (PMC4517649; doi:10.1186/s12889-015-1992-y)
Supplement: Additional file 5: — Distribution of the percentage of DAs within each quintile of Canada wide SES index according to province and territory (p < 0.001 using Pearson chi-square), absolute numbers of DAs are indicated in brackets. (AB=Alberta, BC=British Columbia, MB=Manitoba, NB=New Brunswick, NL=Newfoundland and Labrador, NS=Nova Scotia, NT=Northwest Territories, NU=Nunavut, ON=Ontario, PE=Prince Edward Island, QC=Quebec, SK=Saskatchewan, YT=Yukon). [file 12889_2015_1992_MOESM5_ESM.docx]

|  | | quintile | | | | | Total |
| --- | --- | --- | --- | --- | --- | --- | --- |
|  |  | 1.00 | 2.00 | 3.00 | 4.00 | 5.00 |  |
| Province | AB | 14.6% (763) | 17.8% (932) | 19.7% (1028) | 21.4% (1118) | 26.4% (1381) | 5222 |
|  | BC | 22.3%  (1557) | 19.1%  (1333) | 19.7%  (1372) | 20.9%  (1455) | 18.1%  (1260) | 6977 |
|  | MB | 24.6%  (508) | 18.6%  (383) | 20.1%  (415) | 18.9%  (390) | 17.8%  (368) | 2064 |
|  | NB | 10.5%  (147) | 24.1%  (338) | 23.1%  (324) | 23.8%  (333) | 18.5%  (260) | 1402 |
|  | NL | 25.4%  (262) | 36.9%  (381) | 19.7%  (203) | 12.5%  (129) | 5.5%  (57) | 1032 |
|  | NS | 10.3%  (165) | 20.0%  (322) | 27.1%  (436) | 24.6%  (396) | 18.0%  (290) | 1609 |
|  | NT | 50%  (44) | 15%  (12) | 10%  (8) | 10%  (8) | 10%  (8) | 80 |
|  | NU | 93.9%  (31) | 3.03%  (1) | 3.03%  (1) | 0%  (0) | 0%  (0) | 33 |
|  | ON | 19.0%  (3597) | 17.0%  (3222) | 17.6%  (3338) | 20.0%  (3790) | 26.3%  (4975) | 18922 |
|  | PE | 5.54%  (16) | 15.6%  (45) | 17.6%  (51) | 31.1%  (90) | 30.1%  (87) | 289 |
|  | QC | 24.3%  (3192) | 25.7%  (3377) | 22.7%  (2992) | 17.1%  (2246) | 10.3%  (1355) | 13162 |
|  | SK | 17.5%  (369) | 17.4%  (368) | 21.1%  (447) | 20.9%  (441) | 23.1%  (489) | 2114 |
|  | YT | 25.4%  (17) | 16.4%  (11) | 17.9%  (12) | 17.9%  (12) | 22.4%  (15) | 67 |
| Total | | **10668** | **10725** | **10627** | **10408** | **10545** | **52973** |

Supplement 5. Distribution of the percentage of DAs within each quintile of Canada wide SES index according to province and territory (p<0.001 using Pearson chi-square), absolute numbers of DAs are indicated in brackets. (AB=Alberta, BC=British Columbia, MB=Manitoba, NB=New Brunswick, NL=Newfoundland and Labrador, NS=Nova Scotia, NT=Northwest Territories, NU=Nunavut, ON=Ontario, PE=Prince Edward Island, QC=Quebec, SK=Saskatchewan, YT=Yukon)
